# Oscillating flower colour changes of *Causonis japonica* (Thunb.) Raf. (Vitaceae) linked to sexual phase changes

**DOI:** 10.1038/s41598-022-24252-z

**Published:** 2022-12-01

**Authors:** Y. Furukawa, H. Tsukaya, N. Kawakubo

**Affiliations:** 1 Faculty of Applied Biological Sciences, Gifu University, Gifu, Japan; 2 Graduate School of Science, The University of Tokyo, Tokyo, 113-0033 Japan

**Keywords:** Ecophysiology, Plant ecology

## Abstract

Flower colour change may represent an ‘honest signal’ for pollinators, denoting flowers with good conditions for rewards and pollination. All previously reported flower colour changes are unidirectional, except for an incomplete case in one Fabaceae species. In this study, we discovered a very rare example of complete oscillating flower colour change associated with sexual phase changes in *Causonis japonica* (Vitaceae). More specifically, flower discs of *C. japonica* exhibit an orange colour in the initial male phase then soon fade into pink with desorption of the stamens. Several hours later in the daytime of the same or the following day, with stigma maturation and style elongation, the orange flower disc colour is recovered before fading into pink again. Importantly, we found that the colour change is caused by the accumulation and the degradation of carotenoids. Moreover, nectar secretion was roughly correlated with the abovementioned colour changes. This is the first example of an apparent oscillating colour change mediated by carotenoid content alteration in flowers.

Flower colour change with flower age is widely observed in more than 450 species of many families of angiosperms. Such changes are generally believed to represent a service to pollinators, particularly insects, as they enhance the attractiveness of flowers at the rewarding stage for pollinators, which is also beneficial for pollination^1^ (reviewed in^2^). Whether flower colour changes occur simply as a service to pollinators has been actively debated (e.g.,^2^). Recently, Ito et al.^3^ mathematically proved that such ‘honest signals’ are evolutionarily stable only when flowers are visited by pollinators with both high and low learning abilities. Moreover, temporal flower colour changes are generally unidirectional with time; thus, reversal changes have never been observed^4^. One exceptional case, in *Desmodium setigerum* E. Mey. (Fabaceae), which exhibits a flower colour shift from lilac to turquoise after pollination, indicated a partial flower colour reversal from turquoise to purple when flowers received little or no pollen^5^. Therefore, understanding the variations in flower colour changes is a key aspect of pollination ecology.

*Causonis japonica* (Thunb.) Raf. (= *Cayratia japonica* (Thunb.) Gagnep.) is a perennial vine weed that is widely distributed from the temperate to tropical zones of East Asia, Southeast Asia, India, and Australia^6^. In Japan, this species develops protandrous flowers (ca. 5 mm in diameter) from June to August^7,8^ and is often a target of weed control in agriculture. A previous work reported that *C. japonica* secretes nectar twice a day and that bagging prohibited fruiting, indicating that that it requires pollinators to bear fruits^7^. Many insect species, including ants and wasps, were reported to visit *C. japonica* flowers; among the 121 collected visitors, two individuals of *Apis cerana* were confirmed to carry pollen grains^7^. Based on the above observations, Kakutani et al.^7^ postulated that the diurnal nectar secretion might be synchronized with the flower visiting pattern of *A. cerana.* They also described that flower disc colour of *C. japonica* changed from red to “orange or pink”; therefore, this flower colour change was assumed to be unidirectional^7^. However, during field observations, one of the authors (YF) found that the flower disc colour change of *C. japonica* is not unidirectional. Here, we describe the first case of complete flower colour recovery via a reversal colour change correlated with the sexual phase or with nector production, and demonstrate that this colour change is due to altered carotenoid contents. We also discuss that this oscillating colour change might represent a typical ‘honest signal’ for pollinators.

## Results

Time-course observations on 43 flowers of *Causonis japonica* revealed changes in flower disc colour and sexual expression (Table 1). Temporal changes in floral features showed no difference between diploid (19 flowers) and triploid (24 flowers) individuals. For example, flowering onset times did not differ substantially between ploidy level (diploid: from 07:07 to 13:27, triploid: from 06:58 to 14:49). However, the flowering duration varied significantly from flower to flower, ranging from a minimum of one day to a maximum of six days. Regardless of the ploidy level, all flowers with damaged styles (14 flowers) exhibited brown stigmas after the male phase, then ceased floral development prior to the female phase.

**Table 1:** Characteristics of 43 flowers of *Causonis japonica*.

Flower ID	Inflorescence (plant)	Ploidy	Flower open (male phase)		Flowering period (days)	Style	Female phase		Nectar (total μl)	Colour change O: orange, P: pink
1	a	Diploid	2016/8/8	7:07	5	Matured	Day2	10:52	2.72	O–P–O–P
2	a	Diploid	2016/8/8	7:07	4	Matured	Day2	13:40	1.12	O–P–O–P
3	a	Diploid	2016/8/8	7:07	3	Damaged	–	–	2.87	O–P
4	b	Diploid	2016/8/8	7:12	3	Damaged	–	–	0.74	O–P
5	b	Diploid	2016/8/8	7:12	4	Damaged	–	–	0.85	O–P
6	b	Diploid	2016/8/8	7:12	3	Damaged	–	–	1.75	O–P
7	c	Diploid	2016/8/29	13:12	5	Matured	Day2	9:09	1.48	O–P–O–P
8	c	Diploid	2016/8/29	13:12	5	Matured	Day2	9:09	1.42	O–P–O–P
9	c	Diploid	2016/8/30	13:12	5	Matured	Day2	10:52	1.62	O–P–O–P
10	d	Diploid	2016/8/31	13:27	6	Matured	Day2	11:03	0.98	O–P–O–P
11	d	Diploid	2016/8/29	13:27	5	Matured	Day2	11:03	1.45	O–P–O–P
12	d	Diploid	2016/8/29	13:27	3	Matured	day2	11:03	1.32	O–P–O–P
13	e	Diploid	2016/9/2	9:51	6	Matured	Day1	14:35	0.30	O–P–O–P
14	e	Diploid	2016/9/2	9:51	4	Matured	Day1	14:35	0.13	O–P–O–P
15	f	Diploid	2016/9/2	10:43	4	Damaged	–	–	1.57	O–P
16	f	Diploid	2016/9/2	10:43	4	Damaged	–	–	0.66	O–P
17	f	Diploid	2016/9/2	10:43	5	Damaged	–	–	0.99	O–P
18	^g^	Diploid	2016/9/2	9:12	4	Matured	Day1	15:00	1.05	O–P–O–P
19	^g^	Diploid	2016/9/2	9:12	4	Matured	Day1	15:00	0.93	O–P–O–P
20	h	Triploid	2015/8/1	8:22	4	Matured	Day1	11:22	13.10	O–P–O–P
21	h	Triploid	2015/8/5	9:55	3	Matured	Day1	15:23	6.82	O–P–O–P
22	i	Triploid	2015/8/11	10:35	4	Matured	Day2	10:09	0.50	O–P–O–P
23	i	Triploid	2015/8/11	10:40	4	Matured	Day2	10:15	3.02	O–P–O–P
24	i	Triploid	2015/8/11	12:03	4	Matured	Day2	10:20	3.34	O–P–O–P
25	J	Triploid	2015/8/15	12:12	1	Damaged	–	–	0.29	O–P
26	k	Triploid	2015/8/15	12:16	1	Damaged	–	–	3.88	O–P
27	k	Triploid	2015/8/15	12:17	1	Damaged	–	–	1.72	O–P
28	l	Triploid	2015/8/15	11:27	1	Damaged	–	–	5.20	O–P
29	l	Triploid	2015/8/15	11:28	1	Damaged	–	–	6.70	O–P
30	l	Triploid	2015/8/19	11:30	3	Damaged	–	–	1.69	O–P
31	l	Triploid	2015/8/20	11:42	1	Damaged	–	–	7.53	O–P
32	m	Triploid	2015/8/19	14:14	3	Matured	Day2	11:10	11.55	O–P–O–P
33	m	Triploid	2015/8/20	13:50	3	Matured	Day2	13:20	11.71	O–P–O–P
34	m	Triploid	2015/8/20	14:49	3	Matured	Day2	12:05	14.65	O–P–O–P
35*	m	Triploid	2015/8/20	14:49	4	Matured	Day2	12:05	11.23	O–P–O–P
36	n	Triploid	2015/8/19	13:54	4	Matured	Day2	12:38	9.58	O–P–O–P
37	o	Triploid	2015/8/20	14:49	4	Matured	Day2	12:10	19.31	O–P–O–P
38	P	Triploid	2016/8/8	6:58	3	Matured	Day1	14:09	4.73	O–P–O–P
39	P	Triploid	2016/8/8	6:58	3	Matured	Day1	14:09	5.43	O–P–O–P
40	P	Triploid	2016/8/9	6:58	1	Matured	Day1	14:09	5.33	O–P–O–P
41	q	Triploid	2016/8/8	7:00	4	Matured	Day1	14:21	2.39	O–P–O–P
42	q	Triploid	2016/8/8	7:00	4	Matured	Day1	14:21	1.05	O–P–O–P
43	q	Triploid	2016/8/8	7:00	4	Matured	Day1	14:21	1.31	O–P–O–P

**Figure 1:**
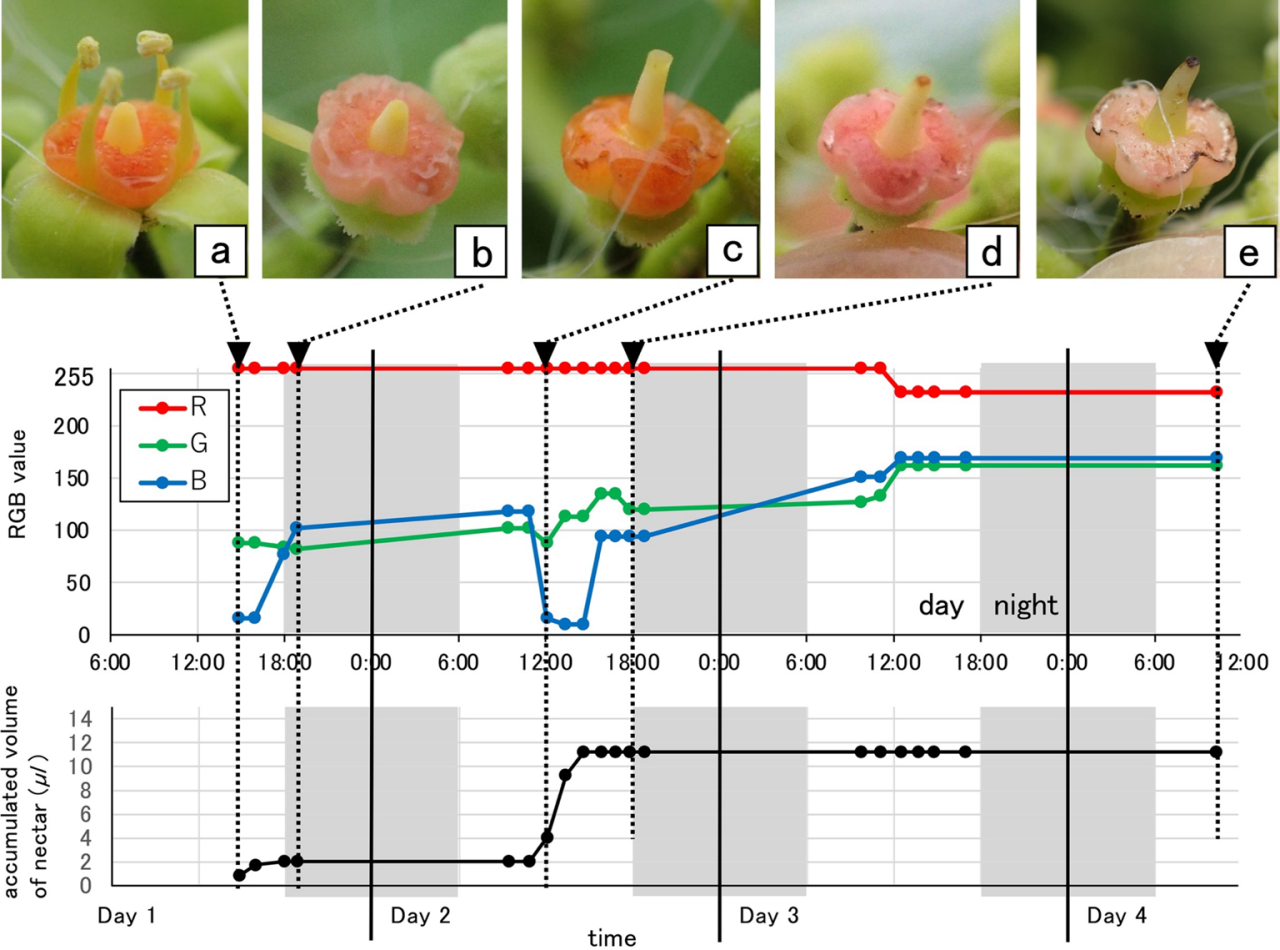
Colour change process of a flower disc in *Causonis japonica* (flower ID 35 in Table 1). Disc colour changed from orange (**a**, RGB: 255, 88, 16) to pink (**b**, RGB: 255, 82, 102) before recovering to orange (**c**, RGB: 255, 88, 16) again, then pink (**d**, RGB: 255, 120, 94) again. In the last stage, the flower disc turned brownish pink (**e**, RGB: 232, 162, 169) then fell off. The two orange colour stages were synchronised with flower sexual activity. (**a**) First orange stage shows stamen activity (male phase); (**c**) second orange stage indicates stigma maturation (female phase). Nectar secretion was active only in the orange stages and more active in the female phase; the same tendency was observed in the other cases shown in Table 1.

Flowers in the same inflorescence are denoted by the same lowercase letter. Colour change process of the flower disc of flower ID 35* is shown in Fig. 1.

Figure 1 shows the typical time-course changes of *C. japonica* flower features (flower ID 35 in Table 1) according to the RGB values (representing the activities of nectar secretion: see below). As in the other 42 examined cases shown in Table 1, the initial colour of this flower disc immediately after anthesis (male phase) was orange (Stage 1, Fig. 1a, RGB: 255, 88, 16), as reported earlier^7^. Immediately after the petals and stamens fell off, the flower disc colour changed to pink (Stage 2, Fig. 1b, RGB: 255, 82, 102). The styles were not yet elongated at this stage, and the flowers were asexual. In 12 cases with damaged styles and brown stigmas, the flower discs remained pink until the flowers fell off (shown as “O–P” in Table 1).

However, in the remaining 31 flowers with normally elongated styles, maturation of the styles (female phase) coincided with the flower discs again exhibiting a distinct orange colour (Stage 3, Fig. 1c, RGB: 255, 88, 16). After the female phase, the flower discs turned pink again (Stage 4, shown as “O–P–O–P” in Table 1), and brownish colouration appeared in the stigmas (Fig. 1d, RGB: 255, 120, 94). Finally, the flower discs turned to a faded pink (Fig. 1e, RGB: 232, 162, 169) just before the flowers fell off. Therefore, the above observations imply that colour-change has a strict correlation with sexual phase.

**Figure 2:**
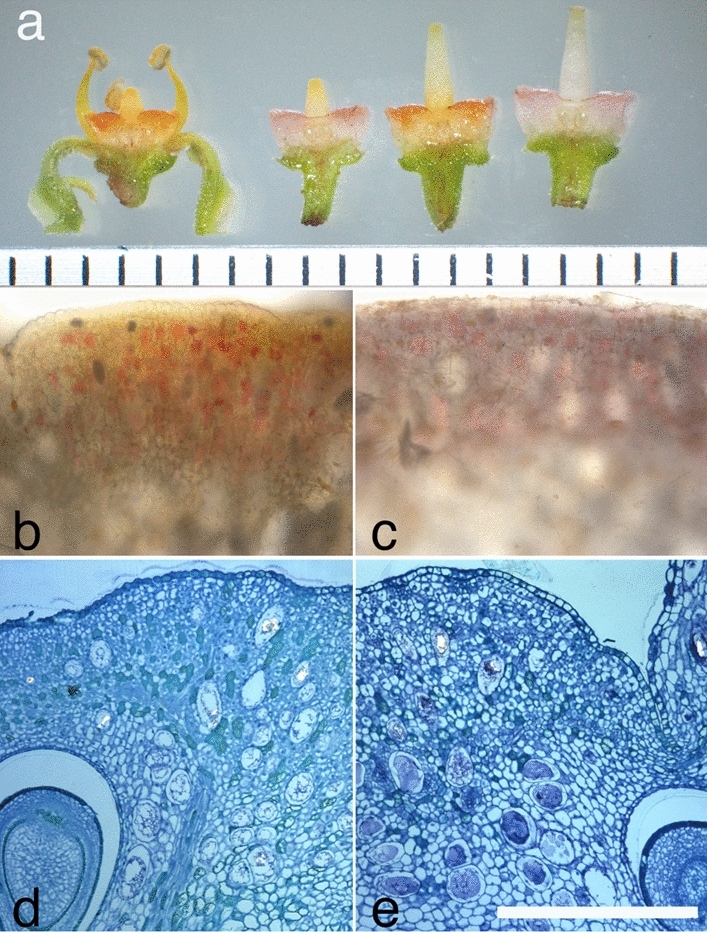
Histology of floral discs of *C. japonica.* (**a**) Floral disc colour change observed in a triploid individual. Flowers were hand-sectioned along the longitudinal axis to show inside colouration of floral disc. Floral phase was judged from the stigma length and colour of the stigma tip; from left, initial stage with orange floral disc and short style, first pink stage with short style, second orange stage with elongated style with matured stigma, and second orange stage with elongated style. Unit of scale bar = 1 mm. (**b**–**e**) Longitudinal sections of floral discs in the initial orange stage (**b**, **d**) and pink stage (**c**, **e**). Scale bar = 500 µm. (**b**, **c**) Hand sections of living floral discs showing pigmentation of vacuoles in some scattered cells. (**d**, **e**) Resin-embedded sections of floral discs showing histology.

The timings of the disc colour change to the second orange stage (female phase) varied depending on the onset time of each flower. Most flowers that opened before 10:00 reached the second orange stage (female phase) on the afternoon of the same day (except for two flowers, ID 1 and 2 in Table 1). Conversely, flowers that bloomed after 10:00 reached the second orange stage (female phase) at approximately noon the following day. These flowering processes were not fully synchronised in the same inflorescence; therefore, pink and orange discs often coexisted in the same inflorescence. Indeed, we can collect various stages of flowers at a time point from one population as shown in Fig. 2a.

Figure 1 also shows the typical time-course changes of nectar activities (flower ID 35 in Table 1), which indicates active nectar secretions during both orange colour stages. That is, the flower discs secreted nectar in both male and female phases, with no visible nectar secretion in the pink stages. Moreover, nectar secretion in the female phase of this flower was higher than that in the male phase, a tendency that was also observed in other flowers; however, the total volume of nectar varied among the flowers shown in Table 1. During anthesis, we confirmed that bees, wasps, ants and other insects visited the flowers as previously described^7^ (Supplementary Fig. 1).

Longitudinal sections of flowers in the pink-coloured and orange-coloured stages (Fig. 2a) revealed that pigmentation occurred only in a subset of parenchymatous cells in both cases (Fig. 2b, c). No structural or cytological changes were observed between the initial orange stage and the pink stages (Fig. 2d, e), suggesting that the observed oscillating colour change depends on the degradation and biosynthesis of orange pigments.

To understand what pigments are involved in the dual colour change of the *C. japonica* flower disc, we extracted carotenoids and chlorophyll with Acetone. Anthocyanin was also extracted with Methanol-HCl. As a result, while anthocyanin content was not significantly altered throughout the stages examined, we found that carotenoid level strongly correlated with the colour change detected by naked eye. More specifically, in stages 1 and 3 the carotenoid content was high (63.8 and 65.3 µg/g dry weight, respectively), but significantly decreased in stages 2 and 4 (14.3 and 36.5 µg/g dry weight, respectively) (Table 2). Interestingly, an increase in chlorophyll content was confined to stage 4 (Table 2). Together, the observed dual colour change was ascribed to the decrease (stage 2) and the increase (stage 3) of carotenoid contents in the flower discs.

**Table 2:** Chlorophyll and Carotenoid contents in the flower discs of *C. japonica*.

	Chl a	Chl b	Carotenoid	N
Stage 1	6.2 ± 0.6	8.2 ± 0.5	63.8 ± 20.8	4
Stage 2	6.9 ± 1.8	7.6 ± 1.0	13.4 ± 3.5	4
Stage 3	4.1 ± 0.2	6.6 ± 0.8	65.3 ± 15.7	3
Stage 4	74.4 ± 27.2	29.3 ± 8.1	36.6 ± 13.7	3

Unit: µg/g dry weight.

## Discussion

In this study, we discovered the first example of complete flower colour recovery associated with sexual phase in *Causonis japonica.* Pigment quantification indicated that the colour changes are correlated with degradation and resynthesis of carotenoid within a short period. This phenomenon itself is astonishing. To date, and to the best of our knowledge, such rapid and repeated cycle of synthesis and degradation of carotenoid has not been recognized. This phenomenon is, on the other hand, highly reasonable if we assume that the orange flower disc displays a reward to pollinators that results in selected visits to flowers in sexually active phases. Indeed the colour change is also correlated with nectar production although the total nectar amount varied among flowers. This is, at least in part, due to environmental factors. For instance, atmospheric humidity has been previously reported to severely affect nectar volume^7^. Hence, it can be said that the presence/absence of nectar is strongly correlated with sexual/asexual phases and also with floral disc colour changes. Past report identified *Apis cerana* as the meaningful pollinating insect of *Causonis japonica*^7^. Because this bee has a highly sensitive blue photoreceptor against *ca.* 450 nm wavelength that corresponds to the absorption peak of carotenoids^9^, it is highly plausible that bees can recognize the aforementioned colour changes. But whether the colour differences are discriminated by floral visitors in a strict sense is not yet examined and is a future issue. Nectar secretion was most active in the male and female phases (^7^ and this study). High nectar secretion in the female phase suggests that nectar may be more important as a reward for pollination of this species because no pollen grain is offered as a reward. Indeed, most active visitors were wasps, which prefer nectar far more than pollen grains^7^. This phenomenon might represent a type of mimicry between male and female flowers that is typically observed in the genus *Begonia*^10^ (reviewed in^2,11^). In this case, it can be understood as sexual mimicry in dichogamy. Anyway, it is necessary to examine the difference in the spectral reflections throughout the bee-visible spectrum including UV among four phases, and to test whether the pollinators recognize the flower colour changes as a strong cue to distinguish nectar-rich flowers at sexual stages from nectar-poor flowers at asexual stages in the near future by bee behavioral analyses.

Interestingly, although all flowers of diploid and triploid individuals investigated in this study exhibited essentially the same process, the second disc colour change was not observed in several flowers that were accidentally damaged, for example, by wind. This might indicate that the second colour change (activation of carotenoid biosynthesis) is physiologically linked to pistil maturation. An important topic for future research is determining whether this link is mediated by phytohormones.

What is the mechanism of the oscillating colour change in *C. japonica* flower discs? Histological and pigment quantitative-based analyses indicated that the flower colour disc changes were caused by the degradation (from orange to pink) and re-biosynthesis (from pink to orange) of carotenoids in a subset of parenchymatous cells. These colour changes occurred over approximately one day; therefore, rapid repression and reactivation of carotenoid biosynthesis/degradation enzymes are speculated as the cause. Furthermore, because plant pigmentation is regulated at the transcriptional level in general (e.g.,^12^), some mRNA species of a key enzyme in the biosynthesis/degradation of carotenoids would be regulated in concert with the sexual phases in *C. japonica.* In angiosperms, many carotenoid biosynthesis/degradation genes have been identified recently. Interestingly, the activity of carotenoid cleavage dioxygenases (CCDs) is known to be responsible for colour polymorphism of many plant species. For example, CCDs activity prescribes the difference of fruit flesh colour between white and yellow peach^13^. It is also known that some azalea and petunia cultivars highly accumulate carotenoid in young floral buds, but later in anthesis carotenoid disappears. This was found to be regulated by CCD4 expression at later stage of floral bud development^14,15^. Thus, examination of CCDs expression levels in floral discs of *Causonis japonica* is one of the future directions.

In this study, we discovered a new type of flower colour change in both diploid and triploid strains of *C. japonica* distributed on Honshu Island, Japan. Previously, we revealed that many haplotypes of Asian *C. japonica* exhibit morphological differentiation; these haplotypes have mutually hybridised to result in some hybrids with low fertility^6^. The most abundant individual on Honshu Island is the triploid line, with most biological research on *C. japonica* conducted on this triploid. Several morphological types are recognised in Japan, with some populations found in the Ryukyu Islands named as *C. tenuifolia* (Wight & Arn.) Gagnep., which are characterised by yellow flower disc and fruits with a flattened obpyriform^16–18^ (Supplemental Fig. 2). These morphological types are all involved in the *Cayratia japonica–Cayratia tenuifolia* species complex (or *Causonia japonica–Causonia tenuifolia* species complex)^6^, which is distributed widely from the temperate to tropical regions of East Asia, Southeast Asia, India, and Australia, with many halotypes differentiated.

As described by Ishikawa et al.^6^ and Okada et al.^16^, the colour of the flower disc immediately after anthesis in the male phase varies among haplotypes (Supplemental Fig. 1). In China and Indonesia, it can be yellow, orange, or red; in Australia, it is lime green (Supplemental Fig. 1). Most haplotypes seem to fix their floral disc colour, suggesting that the oscillating colour change reported in this study occurs only in particular haplotypes of *C. japonica.* Here, we focused on triploid and diploid strains found on Honshu Island that exhibit red–orange or orange flower discs in the male phase. Interestingly, closely related *C. maritima* (Jacks) Jacks and *Cayratia yoshimurai* (Makino) Suess. & Suess. have white and green flower discs, respectively (Supplemental Fig. 2). Because these two species were revealed to be basal against all other *C. japonica* haplotypes^6^, ancestral flower disc colour of *C. japonica* is speculated to be white or green. Taking this into account, the orange-coloured flower disc display is a novel trait that have probably evolved during the diversification of *C. japonica-tenuifolia* species, whereby the acquisition of periodical carotenoid synthesis, degradation and resynthesis in the flower discs is an evolutionary innovation. Therefore, future studies should analyse other haplotypes with different flower disc colours in order to reveal the evolutionary processes of complete flower colour changes in the *C. japonica–C. tenuifolia* species complex.

## Methods

### Samples

Because *C. japonica* is often regarded as a target of weed control in agricultural fields, no conservation regulation is applied to this species in Japan. But all the experiments were performed in accordance with relevant guidelines and regulations. According to previous literature^7^, the flowers first develop an orange or red flower disc in the male phase, which lasts only 2 h. The flowers then lose their stamens and petals and change their disc colour to orange or pink. Finally, several hours after the beginning of anthesis, the flowers shift into the female phase with an elongated style and a matured stigma^7^. In this species, diploid (2n = 40) and triploid (2n = 60) plants often coexist^13^. Therefore, we used flowers (or inflorescences) from both diploid and triploid individuals selected at intervals of 10 m or more because vegetative reproduction by the roots of this species is known to be vigorous^16^.

### Time-course observations

To analyse temporal colour changes of the flower discs, we conducted time-course observations in the field on 43 flowers (19 flowers of seven diploid individuals and 24 flowers of eight triploid individuals) using both the naked eye and digital cameras at Gifu University campus (Gifu city, Gifu Prefecture, Japan). Field observations of the flowers were conducted in two seasons (from 12th July 2015 to 24th August 2015 and from 8th August to 8th October 2016). In addition, observations of the flowers of three triploid individuals collected in Kakamura city, Kanagawa Prefecture, Japan, were performed in a room after cutting during August 2021.

Time-lapse photographic records were performed approximately every 20 min using a digital camera (STYLUS TG-3, Olympus, Tokyo, Japan). Colours were calibrated using the sample sheets of the DIC Colour Guide (19th ed. 2008)^19^. Flower disc colour was evaluated via visual comparison with the DIC Colour Guide sheets for conversion into RGB values (RGB triplet: red, green, blue).

Time-course nectar volume measurements were conducted on the same individuals at Gifu University campus using micro-capillary tubes (Drummond MIUROCAPS 1μL or HIRSCHMANN ringcaps 1.2.3.4.5 μL).

### Histological observations of flower discs

Histological observations were conducted for flowers collected in Hongo campus of the University of Tokyo (Tokyo, Japan) in 2022, by embedding samples in Technovit 7100 resin (Heraeus Kulzer, Hanau, Germany) after fixation with FAA [5% (v/v) acetic acid, 45% (v/v) EtOH, and 5% (v/v) formalin] overnight (or longer) and sequential dehydration with ethanol, as described in previous literature^20^. Resin-embedded specimens were sliced by Leica Rotary Microtome (Leica microsystems, Wetzlar, Germany). 3–6 μm thick histological sections of were placed on cover slips, stained with 0.1% (w/v) toluidine blue in 0.1 M phosphate buffer (pH 7.0), dried, and mounted on glass slides with Entellan new rapid mounting medium (EMD, Millipore, USA). Microscopic observations were performed using a light microscope (LEICA DM4500 B; Leica microsystems).

### Pigment analysis

Flower discs of *C. japonica* were sliced out from each flower with razor blades. Each sample set included more than 30 flower discs. Samples were freeze-dried overnight by FDU-2100 Freeze drier (EYELA, Tokyo, Japan), and then crashed into powder with plastic pestle. Pigment extraction was carried out on 0.01-g dried powder for each sample set with 0.5 mL acetone. After agitation under room temperature for more than 15 min, samples were centrifuged and supernatants were used for spectrophotometry measurements (Hitachi U-3010, Tokyo, Japan). Scan for spectrophotometry was done from 350 to 800 nm; calculation of chlorophyll a/b and carotenoid was done after Wellburn^21^.

## Supplementary Information

Supplementary Information.

## Data Availability

Data are available on request from the authors (HT and KN).
