# Supplementary material for: Oscillating flower colour changes of Causonis japonica (Thunb.) Raf. (Vitaceae) linked to sexual phase changes
Source: Sci Rep. 2022 Dec 1;12:19682. doi: 10.1038/s41598-022-24252-z (PMC9715941; doi:10.1038/s41598-022-24252-z)
Supplement: Supplementary file 1 — Supplementary Information. [file 41598_2022_24252_MOESM1_ESM.pdf]

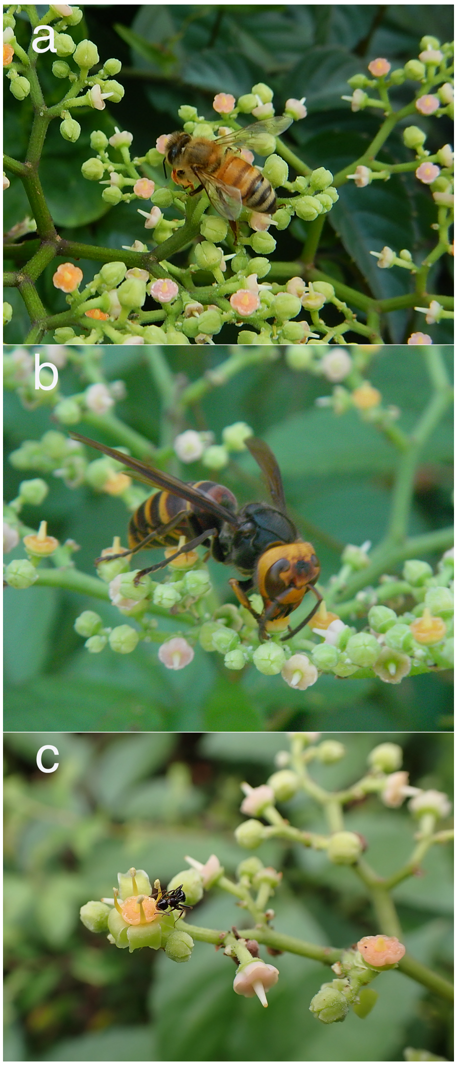

Supplemental Figure 1

Representative insects that frequently visited *Causonis japonica* flowers

Bees (a), wasps (b) and ants (c) were mostly found to collect nector from the flowers. Photographs were taken in Hongo, Bunkyo-ku, Tokyo (a, in 4th August, 2016; b, in 14th August, 2010) and in Kyoto (c, 17th September, 2022). Note all these insects focused on orange-coloured flowers.

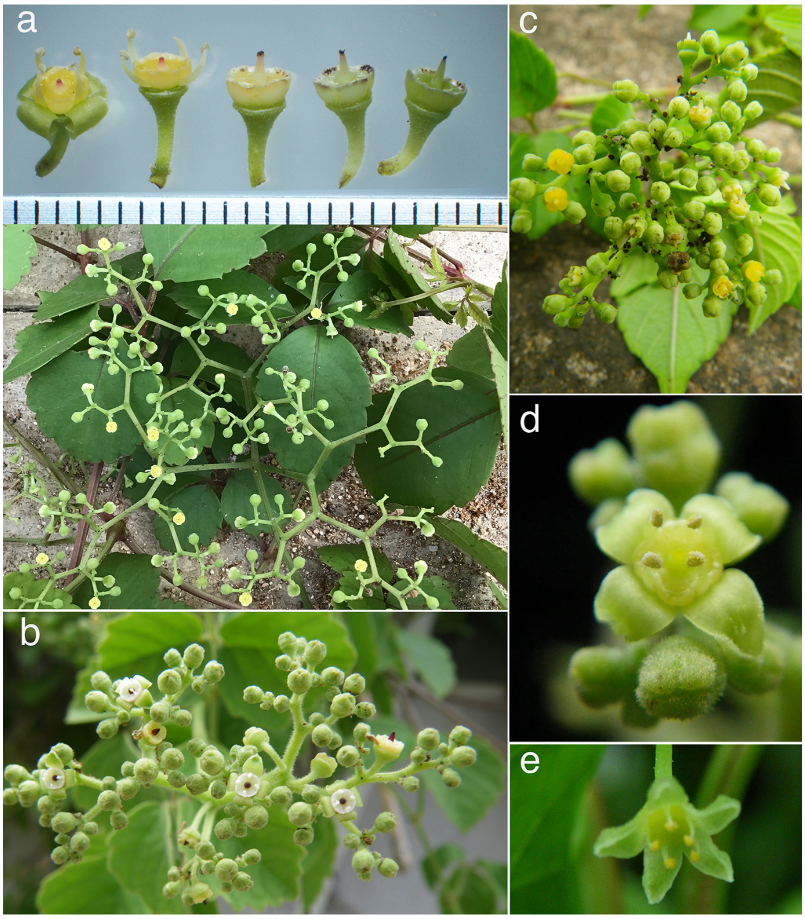

Supplemental Figure 2. Floral disc colour of the closely related species or the other haplotypes of *Causonis japonica* in the other countries

(a) Flowers and inflorescence of *C. tenuifolia*. From left to right the flower stage proceeds. Unit of scale = 1 mm. Note no recovery of flower disc colour; change of the stigma colour; and increase of chlorophyll. (b) White-coloured flower disc of *C. maritima* (Jacks) Jacks. Slight increase of yellowish pigment is seen in older flower. (c) Yellow-coloured flower dics of Indonesian individual of *C. japonica.* (d) Pale-green flower disc of Australian individual of *C. japonica.* (e) Green-coloured flower disc of *Cayratia yoshimurai* (Makino) Suess. & Suess. See Ishikawa et al. (2014) on the detailed information of these materials including haplotype data and molecular phylogeny.
